# Case reports of robot-assisted laparoscopic radical nephrectomy and inferior vena cava tumor thrombectomy: A retrospective analysis

**DOI:** 10.1097/MD.0000000000026886

**Published:** 2021-08-20

**Authors:** Shuaijun Ma, Weijing Jia, Guangdong Hou, Penghe Quan, Longlong Zhang, Xiaozheng Fan, Bo Yang, Xing Su, Jianhua Jiao, Fuli Wang, Jianlin Yuan, Weijun Qin, Xiaojian Yang

**Affiliations:** a Department of Urology, Xijing Hospital, Fourth Military Medical University, Xi’an, China; b Department of Hematology, Xijing Hospital, Fourth Military Medical University, Xi’an, China.

**Keywords:** inferior vena cava, nephrectomy, robot assisted, thrombectomy, tumor thrombus

## Abstract

Renal cell carcinoma is one common type of urologic cancers. It has tendencies to invade into the inferior vena cava (IVC) and usually requires an open surgery procedure. High rates of operative complications and mortality are usually associated with an open surgery procedure. The recently emerged robot-assisted laparoscopic radical nephrectomy (RAL-RN) and IVC tumor thrombectomy have shown to reduce operative related complications in patients with renal cell carcinoma.

This case series study aimed to summarize technical utilization, perioperative outcomes, and efficacies of RAL-RN and IVC tumor thrombectomy in our hospital. A retrospective analysis was performed on clinical data from 20 patients who underwent RAL-RN and IVC tumor thrombectomy from January 2017 to December 2019 in our department.

Patients had a median age of 59 years (interquartile range [IQR], 46–68). Four patients had renal neoplasm on left side and 16 on right side. Nineteen patients underwent RAL-RN (level 0: n = 2) or RAL-RN with IVC thrombectomy (n = 17) (level I: n = 3; level II: n = 12; and level III: n = 3) and 1 patient was converted into an open surgery. The median operative time was 328 minutes (IQR, 221–453). The estimated median blood loss was 500 mL (IQR, 200–1200). The median size of removed renal carcinoma was 67 cm^2^ (IQR, 40–91); the length of IVC tumor thrombus was 5 cm (IQR, 3–7). The postsurgery hospital length of stay was 6 days (IQR, 5–7). The complications included intestinal obstruction (n = 1), lymphatic fistula (n = 1), heart failure (n = 1), and low hemoglobin level (n = 1). The outcomes for patients after 16 months (IQR, 11–21) follow-up were tumor-free (n = 10), tumor progression (n = 4), loss of contact (n = 1), and death (n = 5).

We concluded that RAL-RN and IVC thrombectomy renders good safety profiles including minimal invasiveness, low estimated median blood loss, short hospitalization, low morbidity, and quick renal function recovery. The long-term efficacy needs a further investigation.

## 1 Introduction

Renal cell carcinoma (RCC) is 1 common type of urologic cancers. It accounts for about 2% to 3% of adult malignancies.^[1]^ RCC tends to invade into the inferior vena cava (IVC), which occurs in about 4% to 10% of patients.^[2]^ The surgical management of RCC with levels II to III IVC tumor thrombus, including radical nephrectomy (RN), IVC thrombectomy, and ipsilateral retroperitoneal lymphadenectomy, has been shown to effectively prolong survival rates of patients. However, the complexity of this major open surgical procedure usually results in a complication rate up to 38%, an operative mortality rate of 4% to 10%, and 5-year survival rates of 25% to 65%.^[3–7]^

In order to reduce operative associated complications, robot-assisted laparoscopic RN (RAL-RN) with IVC tumor thrombectomy has recently emerged as a minimally invasive alternative to a conventional open surgical approach.^[8–10]^ This technique offers several benefits for management of RCC with levels II to III IVC tumor thrombus in patients, such as 3-dimensional visualization, precise renal mass removal, reduction of estimated median blood loss (EBL), short hospital length of stay, and similar clinical efficacies, as compared to an open surgical procedure. In this report, we performed a retrospective analysis on clinical data from RCC patients with levels 0 to III IVC tumor thrombus and underwent RAL-RN and IVC tumor thrombectomy. We summarized our clinical experience regarding feasibility, clinical outcomes, and associated complications of this surgical technique.

## 2 Materials and methods

### 2.1 Study population

This retrospective study was approved by the Ethics Committee of Xijing Hospital. Patients have provided informed consent for publication of this case series study. A total of 20 patients who had RCC with levels 0 to III IVC tumor thrombus and underwent RAL-RN and IVC thrombectomy from Jan 2017 to December 2019 in our department were included. The disease duration varied from 6 days to 12 months. The main complains from patients for their initial visits to our department were gross hematuria (n = 6), low back pain (n = 4), and edema in both lower extremities (n = 1). Other patients (n = 9) were diagnosed during regular physical examinations. Seven patients had smoking histories and 1 patient had alcoholism. Other health preconditions in patients included hypertension (n = 6), diabetes (n = 3), and drug-induced hepatotoxicity (n = 1). Patients underwent a standard preoperative procedure and imaging examinations including color Doppler ultrasound, three-dimensional computed tomography , and/or magnetic resonance imaging to determine tumor sizes and locations as well as vascular invasions. Ten patients received preoperative targeted therapies, including sunitinib (37.5 mg, once per day, n = 8) for 1–4 months and sorafenib (400 mg, twice per day, n = 2) for 1 and 5 months, respectively.

### 2.2 Right-sided robot-assisted laparoscopic radical nephrectomy and inferior vena cava thrombectomy

Patient was secured in a 60° left lateral decubitus position for IVC exposure. A disposable 12 mm trocar was placed as the camera port into the abdomen which was upper lateral to rectus abdominis and 3 to 5 cm lateral from umbilicus. Three 8 mm trocars were placed into the following positions for robotic arms: 3 cm below the 12^th^ rib in the right midclavicular line, 8 to 10 cm lateral from the camera port in the upper middle of anterior superior iliac spine, and the level of 8 to 10 cm distance from the camera port and the second robotic arm port and lateral to the lower right rectus abdominis. A monopolar scissor, bipolar Maryland clamp, or Prograsp grasping forceps was connected with the first, second and third robotic arms, respectively. Three assistant ports were placed. The first (5 mm trocar) was below xiphisternum in the mid-ventral line. The second (12 mm trocar) was between the first robotic arm and camera port, and the third (12 mm trocar) was between the third robotic arm and camera port.

For the vena cava control, hepatorenal, hepatocolic, and falciform ligaments were incised, the parietal peritoneum was opened to access interaortocaval region, and the right colon and duodenum were reflected to expose the vena cava and other blood vessels including the lumbar veins, gonadal veins, short hepatic veins, right renal artery, right and left renal veins. The tumor thrombus segment of IVC was fully dissociated under the guidance of laparoscopic ultrasound. For level II tumor thrombus, liver was raised, and 1 to 3 short hepatic veins were ligated. For level III tumor thrombus, left and right side lobes of liver were turned, and 2 to 5 root short hepatic veins were ligated to expose the first and second porta hepatis. The double-loop rubber vascular band was used to clinch the distal, proximal of IVC and left renal vein. Rubber tubes of about 1 cm length were inserted into the vascular bands. The lumbar vein and other branches of IVC were ligated. The right renal artery was double-clipped with Hem-o-lok and then dissociated. The right renal vein was severed by an electric cutting closure. After adequate preparation, the distal end of the IVC, the left renal vein, and the proximal end of the IVC were sequentially blocked. The wall of the IVC was cut open and the tumor thrombus was completely removed. The wall of IVC that was infiltrated by tumor thrombus was partially removed. A 4-0 polypropylene suture was used for caval reconstruction; the IVC lumen was flushed and maintained with heparinized saline before closed. The proximal end of the IVC, left renal vein, and the distal end of IVC were released sequentially to restore caval flow. Before the distal end of the IVC was released, blood oozing and any possible residue tumor thrombus remained in IVC were checked. Right side RAL-RN was performed after IVC thrombectomy. En bloc right adrenalectomy was performed in patients (n = 1) who had metastasis in the right adrenal glands.

### 2.3 Left-sided robot-assisted laparoscopic radical nephrectomy and inferior vena cava thrombectomy

Left renal arterial embolization was performed 1 to 2 hours prior to IVC thrombectomy. The patient position and port replacement were the same as for the right-sided IVC thrombectomy. A sequential clamping of the distal end of the IVC, right renal artery, right renal vein, and the proximal end of IVC were performed. The wall of the IVC was cut open and the tumor thrombus was completely removed, followed by caval wall reconstruction and caval flow restoration. The patient was then converted into a 60° right lateral decubitus position for a left-sided RAL-RN. An adrenalectomy was performed in patients (n = 1) with metastasis in the left adrenal glands.

### 2.4 Postoperative care and follow-up

Routine postoperative care including wound care, complication and functional check, computed tomography or magnetic resonance imaging, and laboratory examinations were performed at 3 to 6 months after operation. The follow-up was performed from 3 to 36 months. Eleven patients received postoperative targeted therapies. Five patients were given oral sunitinib (37.5 mg, once per day). Five patients were given oral sorafenib (400 mg, twice per day). One patient was given oral axitinib (5 mg, twice per day). Two patients who had oral sunitinib or sorafenib initially were given axinitib after the progression of their diseases.

### 2.5 Data presentation

The data was presented as median (interquartile range) or n (%).

## 3 Results

The median age of patients was 59 (46–68) with 13 males and 7 females (Table 1). Four cases had renal neoplasm on left side and 16 on right side. The median size of the RCC mass was 67 cm^2^ (40–91). The median length of the IVC tumor thrombus was 5 cm (3–7). The levels of the IVC tumor thrombus were 2 cases for level 0, 3 cases for level I, 12 cases for level II, and 3 cases for level III (Table 1). Other clinical characteristics is listed in Table 1.

**Table 1:** Patient demographic data and basic characteristics.

Variable	Result, median (IQR), or n (%)
Patients	20
Age (yr)	59 (46–68)
Gender, male/female	13/7
BMI (kg/m^2^)	22 (19–26)
Health conditions	
Smoking	7 (35%)
High blood pressure	6 (30%)
Diabetes	3 (15%)
Drug-induced hepatotoxicity	1 (5%)
Affected kidney	
Left	4 (20%)
Right	16 (80%)
Size of RCC (cm^2^)	67 (40–91)
IVC thrombus classification	
Level 0	2 (5%)
Level I	3 (15%)
Level II	12 (60%)
Level III	3 (15%)
Size of IVC thrombus (cm)	5 (3–7)
Preoperative embolization	3 (15%)
Preexisting metastasis	1 (5%)
Preoperative targeted therapy	10 (50%)

BMI = body mass index, IQR = interquartile range, IVC = inferior vena cava, RCC = renal cell carcinoma.

Nineteen out of 20 patients were successfully underwent RAL-RN with or without IVC thrombectomy. A total of 13 patients had right-sided RAL-RN with IVC thrombectomy. One patient had right-sided RAL-RN with partial IVC excision. Four patients had left-sided RAL-RN with IVC thrombectomy, and 2 patients had right-sided RAL-RN. Only 1 patient was converted into an open surgery. The perioperative data is listed in Table 2. The median operation time was 328 minutes (221, 452) and the average clamping time was 24 minutes (18, 37). The median EBL was 500 mL (200–1200). The postoperation hospital length of stay was 6 days (5–7). The complications included intestinal obstruction (n = 1), lymphatic fistula (n = 1), heart failure (n = 1), and low hemoglobin level (n = 1).

**Table 2:** Perioperative data.

Variable	Result, median (IQR), or n (%)
Patients (n)	20
Operative time (min)	328 (221, 452)
Thrombectomy time (min)	13 (8, 24)
IVC clamp time (min)	24 (18, 37)
Nephrectomy time (min)	50 (30, 118)
EBL (mL)	500 (200–1200)
Patients receiving intraoperative transfusions	9 (45%)
Lymph nodes removed	9 (45%)
Lymph nodes positive	3 (15%)
Complications (n)	4 (20%)
Length of hospital stay after surgery (d)	6 (5–7)
Conversion to open surgery (n)	1 (5%)

EBL = estimated median blood loss, IQR = interquartile range, IVC = inferior vena cava.

The pathological characteristics of tumor cell malignancy and IVC thrombus is listed in Table 3. Pathological diagnoses showed that 9 patients had clear-cell carcinoma, 4 had mixed renal carcinoma, 1 had XP11.2 translocation/TFE3 gene fusion associated with RCC, 2 had RCC, 1 had neuroendocrine carcinoma, 2 had eosinophilic cell tumor, and 1 had renal malignancy with unidentified direction of differentiation.

**Table 3:** Tumor pathological data.

Variable	Result, n (%)
Patients (n)	20
Clinical stage	
T3a	2 (10%)
T3b	13 (65%)
T3c	3 (15%)
T4	2 (10%)
Positive lymph nodes	3 (15%)
Metastasis in adrenal glands	2 (10%)
Clear-cell carcinoma	9 (45%)
Mixed renal carcinoma	4 (20%)
XP11.2 translocation/TFE3 gene fusion associated with renal cell carcinoma	1 (5%)
Renal cell carcinoma	2 (10%)
Neuroendocrine carcinoma	1 (5%)
Eosinophilic cell tumor	2 (10%)
Renal malignancy does not indicate the direction of differentiation	1 (5%)

The patients were followed up from 3 to 36 months. Nine patients showed tumor-free and 3 patients had tumor progression. One patient got loss of contact and 7 died with a median survival time of 14 months (4–16) (Table 4).

**Table 4:** Clinical outcomes for follow-up.

Variable	Result, median (IQR), or n (%)
Patients (n)	20
Follow-up (mo)	16 (12, 21)
Cancer status	
Disease free (n)	9 (45%)
Relapse (n)	3 (15%)
Patient status	
Alive (n)	12 (60%)
Loss of contact (n)	1 (5%)
Dead (n)	7 (35%)
RCC type (n), survival time (mo)	Neuroendocrine carcinoma, 1, 20
	XP11.2 translocation/TFE3 gene fusion associated with RCC, 1, 17
	Renal malignancy does not indicate the direction of differentiation, 1, 16
	Eosinophilic cell tumor, 2, 14, and 24
	RCC, 2, 4, and 3

IQR = interquartile range, RCC = renal cell carcinoma.

## 4 Discussion

RN and IVC tumor thrombectomy are among the most challenging open surgical procedures in oncologic urology. Level III IVC tumor thrombectomy may be associated with up to 38% of major complication rates and result in 4% to 10% of perioperative mortality rates.^[3,4,6]^ In an effort to reduce the operative associated morbidity and improve the oncologic efficacy, robotic-assisted approaches have emerged and practiced in recent years. Particularly, Abaza^[8]^ reported the first series of 5 cases in 2011 using RAL-RN and IVC thrombectomy and Lee and Mucksavage^[11]^ reported their similar experiences in 2012. In 2015, Gill et al^[3]^ reported their series of 16 cases, Wang et al^[7]^ reported their series 17 cases in 2016. These initial studies have provided a very comprehensive experience and initial evaluation of this technique. In this study, we reported 20 cases in this study, like abovementioned authors, showing that RAL-RN and IVC thrombectomy technique renders its major benefits on perioperative safety, technical reproducibility, and comparable clinical outcomes.

Classification of IVC levels is crucial to decide whether to manage tumor thrombi by RAL IVC thrombectomy or an open surgery. We used the numeric levels described by Blute et al^[4]^ in 2004 in which the major hepatic venous confluence is used as the landmark to classify IVC levels. Based on this classification, we had 2 level 0, 3 level I, 12 level II, and 3 level III. In level III, a tumor thrombus extends to hepatic venous confluence which will make RAL–IVC thrombectomy particularly challenging due to a limited anatomic zone for an operation. Case series of level III or IV RAL–IVC thrombectomy have been recently reported.^[6,9,12]^ We also successfully performed 3 cases level III RAL–IVC thrombectomy without major complications, which was a result from precise vascular control and meticulous vascular dissection during operation.

Whether targeted therapy is beneficial to RCC with IVC tumor thrombus is still under debate. Some studies showed that targeted therapy can help metastatic RCC but do little on RCC with IVC tumor thrombus.^[13,14]^ However, tumor embolism in IVC may cause a release of more tumor cells from thrombus into circulation, leading to metastasis. Also, RAL-RN and IVC thrombectomy may result in a local spillage of tumor cells during operation. Therefore, patients have a significant risk of tumor reoccurrence. Furthermore, 5-years cancer-specific survival rate is only about 25% to 65% for patients with RCC involving IVC tumor thrombus after surgery.^[5,10,15]^ Therefore, frontline targeted therapy shall be considered. In our study, preoperative targeted therapy was applied in 10 patients and postoperative targeted therapy was applied in 10 patients according to our institutional experience. However, the related benefit needs to be further investigated and determined.

There are several limitations for this study. First patient cohort is small, particularly only 3 cases of level III. Second, our follow-up time from 3 to 36 months is short. We are unable to access the long-term efficacy of RAL–IVC thrombectomy. Third, patient groups are heterogeneous with complexity of tumor anatomic locations and degree of malignancy, thus leading to variations in perioperative and postoperative data such as operative duration, EBL, length of stay, tumor-free rate, and survival rate. Fourth, whether RAL–IVC will improve 5-year survival rate is yet to be determined. We are unable to compare RAL–IVC thrombectomy with an open surgery side by side or in a retrospective setting due to the short follow-up time; this will be the focus of our future investigation to determine whether 1 approach provides a superior efficacy over the other.

## 5 Conclusion

Collectively, RAL–RN and IVC thrombectomy has been proven to be safe and effective for management of RCC involving IVC. It needs expertise in multidiscipline and renders good safety profiles including minimal invasiveness, low EBL, short hospitalization, low morbidity, and quick renal function recovery. However, its long-term efficacy comparing with an open surgery needs further investigation.

## Author contributions

SM, WJ, and XY contributed to the conceptualization and supervision of this study. SM and WJ performed the surgeries and analyzed the data and made major contributions to the manuscript preparation. LZ, XF, and BY contributed to the data analysis. FW, JY, and WQ contributed to the project administration. All authors have read and approved the final manuscript.

**Conceptualization:** Fuli Wang, Jianlin Yuan, Weijun Qin, Xiaojian Yang.

**Data curation:** Shuaijun Ma, Longlong Zhang.

**Formal analysis:** Longlong Zhang.

**Investigation:** Weijing Jia, Guangdong Hou, Xiaozheng Fan, Xing Su.

**Methodology:** Shuaijun Ma, Guangdong Hou, Xiaojian Yang.

**Project administration:** Penghe Quan, Xiaozheng Fan.

**Resources:** Weijing Jia, Xiaozheng Fan.

**Software:** Shuaijun Ma, Weijing Jia, Guangdong Hou.

**Supervision:** Jianlin Yuan.

**Validation:** Weijing Jia.

**Visualization:** Shuaijun Ma, Penghe Quan, Bo Yang.

**Writing – original draft:** Shuaijun Ma.

**Writing – review & editing:** Penghe Quan, Bo Yang, Jianhua Jiao, Fuli Wang, Weijun Qin, Xiaojian Yang.
